# A Preliminary Study on a Form of the 24-h Recall That Balances Survey Cost and Accuracy, Based on the NCI Method

**DOI:** 10.3390/nu14132740

**Published:** 2022-06-30

**Authors:** Kun Huang, Liyun Zhao, Hongyun Fang, Dongmei Yu, Yuxiang Yang, Zizi Li, Di Mu, Lahong Ju, Shujuan Li, Xue Cheng, Xiaoli Xu, Qiya Guo

**Affiliations:** NHC Key Laboratory of Trace Element Nutrition, National Institute for Nutrition and Health, Chinese Center for Disease Control and Prevention, Beijing 100050, China; 15550807252@163.com (K.H.); zhaoly@ninh.chinacdc.cn (L.Z.); fanghy@ninh.chinacdc.cn (H.F.); yu_dongmei@126.com (D.Y.); yxyang_ninhccdc@126.com (Y.Y.); lizz@ninh.chinacdc.cn (Z.L.); mudi@ninh.chinacdc.cn (D.M.); julh@ninh.chinacdc.cn (L.J.); lisj@ninh.chinacdc.cn (S.L.); chengxue@ninh.chinacdc.cn (X.C.); xuxl@ninh.chinacdc.cn (X.X.)

**Keywords:** 24-h recall, non-consecutive, accuracy, dietary intake, NCI, usual intake

## Abstract

The 24-h recall (24HR) is a short-term dietary assessment instrument that is widely used in large-scale nutrition surveys. The number of survey days is critical in the accuracy of estimates. The multiple, repeated collection of 24HRs can yield reliable dietary intakes, whereas that is not always feasible due to staffing, equipment, financial, and temporal constraints. The NCI (National Cancer Institute) method was developed to address this limitation by using only within-person variance to calculate usual dietary intake. However, the performance of different forms of 24HRs based on the NCI method remains unclear. The aim of this study was to explore a form of 24HR based on the NCI method that can balance accuracy and survey cost. A total of 595 subjects completed 7 consecutive 24HRs in each season, for a total of 28 24HRs. The averages of the 28 collection days were defined as the reference value to compare the performance of 24HRs for two consecutive days (C2), three consecutive days (C3), two non-consecutive days (NC2), and three non-consecutive days (NC3) for estimating the dietary intakes of Chinese adults. The equivalence test was used to evaluate whether the estimates of scenarios NC2 and NC3 were equivalent. Additionally, the accuracy of a scenario of NC2 which included a weekend was compared to that of a scenario of NC2 which included two weekdays. All results of the 24HRs in each scenario were corrected by the NCI method. Bias/relative bias and mean bias/mean relative bias were used as measures of precision and accuracy, respectively. The results showed that the precision was similar among the four scenarios, while the accuracy relationship varied among the different dietary components. In general, scenario NC3 was the most accurate, followed by scenario NC2, which was close to the former. The form using non-consecutive days was more accurate than that using consecutive days, and the main factor affecting the accuracy of the 24HRs was the continuity between multiple survey days rather than the number of days. The means and major percentiles of energy, nutrients, and frequently consumed food in scenarios NC2 and NC3 were functionally identical. The accuracy of the scenario of NC2 which included a weekend was higher than that of scenario NC2, which consisted of only weekdays. The above results indicated that the adoption of two, non-consecutive 24HRs consisting of a weekend and a weekday to collect dietary data prior to correction by the NCI method, is a feasible approach to balancing survey costs and accuracy in large-scale nutrition surveys.

## 1. Introduction

As an important part of a nutrition survey, the results of a dietary survey allow for the assessment of population-level food and nutrient intake and the risks of inadequate or excessive intakes. Additionally, it is an interesting exposure factor in the study of the relationship between diet and chronic diseases. The 24-h recall (24HR), a short-term dietary assessment instrument, requires the investigator to help the subject accurately recall and record the types and amounts of all of the foods consumed in the past 24 h [1]. This method has been widely used in many large-scale nutrition surveys due to the advantages of validity, high response rate, and simplicity [2,3,4,5]. However, it also has some limitations that affect accuracy [6]. Since dietary intake is affected by season, 24HRs conducted in different seasons may yield different results [7,8]. In addition, dietary intake on weekends tends to be higher than that on weekdays, and there may be significant differences in dietary intakes between two adjacent days [9,10]. These limitations lead to different forms of short-term 24HRs with varying levels of performance. A previous study has shown that the number of 24HRs was a major factor in their accuracy, with those conducted on non-consecutive survey days being more accurate than those performed on consecutive survey days, but the level of improvement was small [11]. Various forms of 24HR based on the within-person mean method share common drawbacks, in that they include large errors between the estimated and the true values, and they tend to overestimate low intakes and underestimate high intakes. These errors may result in distorted percentages of subjects below or above specified cut-points [12]. Another important consequence of exaggerated variation is that the relationships between diets and health outcomes will be obscured [13]. Administering enough 24HRs can obtain help to the true daily intake of an individual, but this is difficult to implement in practical investigations [14,15]. Some studies have used the ratio between within-person and between-person variance to calculate the number of survey days needed to estimate the usual intake for certain nutrients [16]. The results showed that it took 3 days to obtain the usual energy intake and 41 days to obtain the usual vitamin A intake [17].

Several similar statistical modelling methods have been proposed to obtain usual intakes by correcting for the measurement errors of short-term 24HRs. These corrected methods include the National Cancer Institute (NCI) method [18], the Multiple Source Method (MSM) [19], the Iowa State University (ISU) method [20], and the Statistical Program to Assess Dietary Exposure (SPADE) method [21]. Among these methods, the NCI method is widely used because it allows for adjustment by covariates, and it allows for weighting to represent the total population [18]. The validity of the NCI method has been verified in many studies, and its estimates are more accurate than those of the within-person mean method [22,23,24,25]. However, studies comparing the performance of various forms of 24HRs calculated by the NCI method, are still lacking.

The aim of this study was to compare the performance of two consecutive 24HRs, three consecutive 24HRs, two non-consecutive 24HRs, and three non-consecutive 24HRs, using the NCI method to generate estimates of dietary intakes and the risks of nutrient deficiencies/excesses. The secondary aim was to determine which of these would be a better form of the 24HR to balance the accuracy with the cost of dietary surveys. The average of 28 24HRs administered in a single year were used as the gold standard for these purposes.

## 2. Materials and Methods

### 2.1. Study Design and Participants

The present study is a follow-up study to one conducted from December 2019 to December 2020. The participants were aged 18 to 60 years and lived in Zhejiang and Shanxi provinces in China. One rural survey site and one urban survey site were selected from each province. The selected survey sites had served as the China Nutrition and Healthy Surveillance 2015 (CNHS 2015) monitoring sites and thus had a basis for conducting dietary surveys. Purposive sampling was used to recruit 198 participants at each survey site, divided in half by gender. A total of 7 consecutive 24HRs were conducted quarterly during the one-year follow-up period. The seasons were divided into Spring (March to May), Summer (June to August), Autumn (September to November), and Winter (December to February). Finally, a total of 780 participants completed 28 24HRs. Detailed descriptions of the sampling and survey protocols are provided in the published article [11].

**Figure 1:**
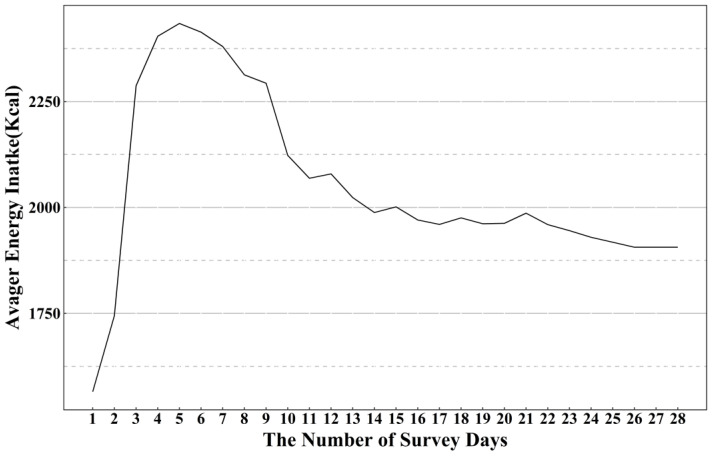
Line graph of an individual’s average energy intake versus the number of survey days.

In the present study, we first removed the survey days with unusual energy intake levels (outside the range of 600 to 4200 kcal per day for males or 400 to 3500 kcal for females) [12]. Then, 28 subjects were excluded for participating in fewer than 23 survey days with reasonable energy intake levels [26]. We also excluded 157 subjects whose average energy intake as calculated over all of the collection days could not represent the usual energy intake of that individual. As shown in Figure 1, as the number of survey days increased, the average energy intake of individuals tended to stabilize. When the relative error between an individual’s average energy intake over the course of 23 to 27 survey days and that of 28 survey days was within 5%, we assumed that the calculation for 28 survey days was the approximation of the usual intake for that individual. If an individual completed a total of 27 24HRs, the percentage differences between the average energy intake calculated for the 22–26 survey days and the 27 survey days were compared. A total of 595 eligible participants were finally included in our study. The characteristics of the participants have been described elsewhere [11].

The study protocol was approved by the Ethics Committee of the Chinese Center for Disease Control and Prevention (No. 201519-B), and all participants signed an informed consent form before participating.

### 2.2. Data Collection and Measurements

The design of the questionnaire for this study was based on the standard questionnaire of the CNHS 2015, which consisted of four domains: basic information, health status, dietary information, and condiment consumption. All the information was collected through face-to-face interviews. Before the investigation, all investigators received standardized training, and quality control was implemented during data collection.

### 2.3. Dietary Intake Assessment

Individual dietary data was recorded through face-to-face household visits by investigators. Participants were asked to complete 7 consecutive 24HRs in each quarter, for a total of 28 survey days. On each visit, trained investigators used food models and picture aids to help participants recall the types, amounts, cooking methods, and locations of all the foods they had consumed in the past 24 h. The face-to-face interviews also recorded whether the respondents were working on each visit day. Daily energy and nutrient intakes for individuals were calculated according to the Chinese Food Composition Tables [27]. For this study, we targeted 33 dietary components that have been evaluated frequently, including energy, 22 nutrients (fat, carbohydrate, protein, dietary fiber, cholesterol, vitamin A, vitamin C, vitamin E, vitamin B1, vitamin B2, vitamin B3, vitamin B9, calcium, iron, zinc, magnesium, sodium, potassium, phosphorus, manganese, copper, and selenium), and 10 food categories (cereals, vegetables, fish, pork, fruits, poultry, tubers, eggs, dairy, and legumes).

### 2.4. Data Sets

According to different forms of administering multiple 24HRs, we divided them into 4 scenarios, including C2 (2 consecutive days), C3 (3 consecutive days), NC2 (2 non-consecutive days), and NC3 (3 non-consecutive days). “Non-consecutive days” were defined as periods in which least one day passed between two adjacent survey days. Seasonal and weekend effects may lead to different results at different survey times. To fully compare the performance between the four scenarios, we generated data sets containing all possible combinations of collection days for the four scenarios. The data sets generated by the 4 scenarios over the 7 consecutive survey days are shown in Table 1. Since the dietary survey was conducted each quarter, scenarios C2, C3, NC2, and NC3 generated 24, 20, 60, and 40 data sets, respectively.

The agreement between the participants’ self-reported work schedules and weekdays as defined by the date was greater than 99%. Therefore, weekdays were defined as being those days between Monday and Friday, and weekends were defined as being Saturday and Sunday.

### 2.5. Statistical Analysis

The true dietary intake was defined as the average intake of all (23 or more) 24HRs for each participant [24]. This was also considered to be the ‘gold standard’ for the usual dietary intake results in further analyses. For each data set of the four scenarios, we estimated the usual intake for selected dietary components using the NCI method.

The NCI method is a correction method created by the National Cancer Institute of the USA to reduce the measurement error found in dietary intake calculations through the removal of intra-individual variability [18]. It is a mixed model composed of two parts, in which the first part uses the logical regression method to estimate the probability of consumption, and the second part uses the mixed effect linear model to estimate the amount of consumption [12]. Means and percentiles for the usual intake for each dietary component were estimated using the MIXTRAN and DISTRIB macros in version 2.1 of the SAS software. As recommended, the amount-only model was used to estimate dietary components with a zero-intake percentage below 5%. If the zero intake of a dietary component exceeded 5%, the correlation model or uncorrelation model was chosen, based on whether there was a correlation between the probability of consumption and the consumption-day amount [28]. Covariates were also included in the model to account for age, gender, and the weekend/weekday effect [29].

To compare the estimated value with the true value, we calculated the bias B for each dietary component,

(1) $$B_{ij}=E_{ij}-T_{ij}$$

mean bias (MB),

(2) $$MB_{ij}=\left|\frac{\sum_{i=1}^{N_{j}}B_{ij}}{N_{j}}\right|$$

relative bias (RB),

(3) $$RB_{ij}=\frac{B_{ij}}{T_{ij}}\times 100\%$$

and mean relative bias (MRB),

(4) $$MRB_{ij}=\left|\frac{\sum_{i=1}^{N_{j}}RB_{ij}}{N_{j}}\right|$$

where $E_{ij}$ and $T_{ij}$ are defined as the estimated and true values of the parameter for the dietary components i and scenario j, respectively, and $N_{j}$ is the number of data sets for scenario j.

To demonstrate the differences between the four scenarios more visibly, the relative bias and the mean relative bias were used to compare dietary components with less than 5% of zero intake, while the bias and mean bias were used for dietary components with greater than 5% of zero intake.

Equivalence testing was used to evaluate whether the parameters between scenario NC2 and NC3 were equivalent. The equivalence margin was defined as 5% of the estimates from scenario NC3 [30]. Confidence intervals of 90%, with a confidence level α equal to 0.05, were calculated as the equivalence testing relative to the 2 one-sided tests [31].

The NCI method and other analyses were conducted using SAS version 9.4 (SAS Institute Inc., Cary, NC, USA), and all plots were constructed using R version 4.1.2.

## 3. Results

### 3.1. Comparison of the Four Scenarios

**Figure 2:**
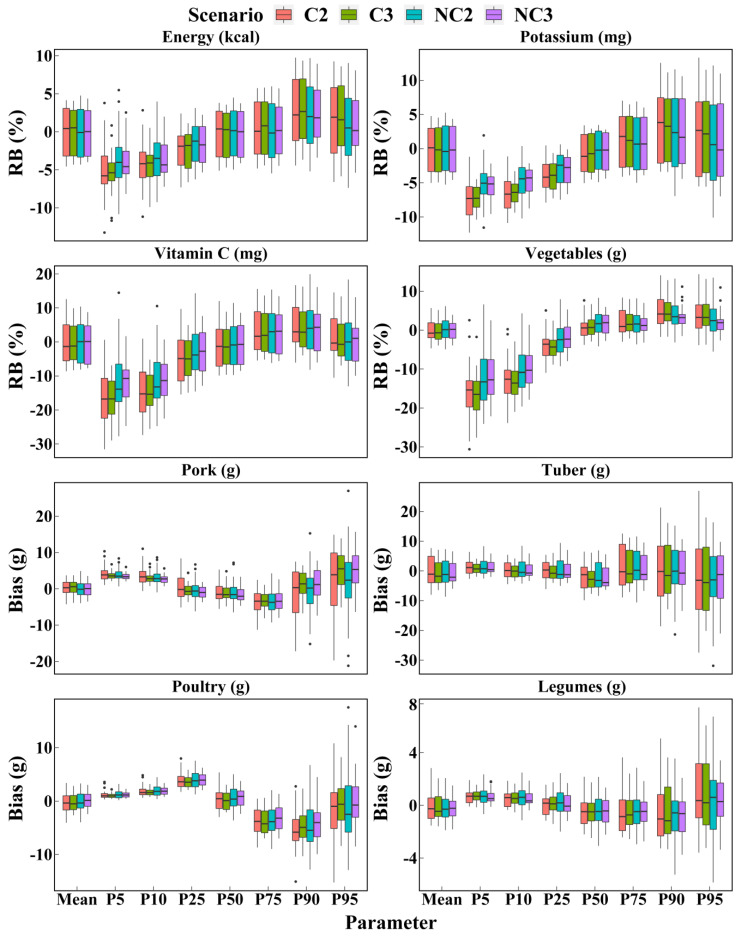
Boxplots of bias and relative bias of intake calculated for the four scenarios based on the NCI method. Boxplots of bias and relative bias for each dietary component calculated for the 4 scenarios are available in Supplementary Figure S1. C2 = 2 consecutive days; C3 = 3 consecutive days; NC2 = 2 non-consecutive days; NC3 = 3 non-consecutive days; RB = Relative bias.

Figure 2 shows the boxplots of the biases and relative biases for 8 dietary components as calculated by the NCI method under the four scenarios. In most cases, the distribution of the bias between the four scenarios was similar. It is interesting to note that, in the four scenarios, the range of the bias from the 5th to 95th percentiles increased, while the range of the bias in the mean was similar that of the median. However, it was clear that the scenarios with non-consecutive days were more accurate than the scenarios with consecutive days for the estimation of the 5th, 10th, 90th, and 95th percentiles across most dietary components. As expected, accuracy was the highest for the estimation of the mean in each scenario.

**Figure 3:**
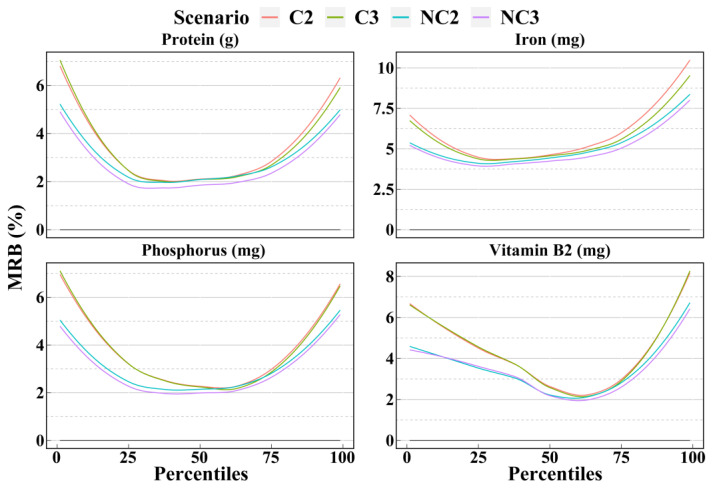
Smooth curves of the percentile (from 1st to 99th) intakes of dietary components in group 1 as calculated by the NCI method based on the 4 scenarios. Smooth curves of bias and relative bias for 12 dietary components calculated for the 4 scenarios are available in Supplementary Figure S2. C2 = 2 consecutive days; C3 = 3 consecutive days; NC2 = 2 non-consecutive days; NC3 = 3 non-consecutive days; MRB = Mean relative bias.

**Figure 4:**
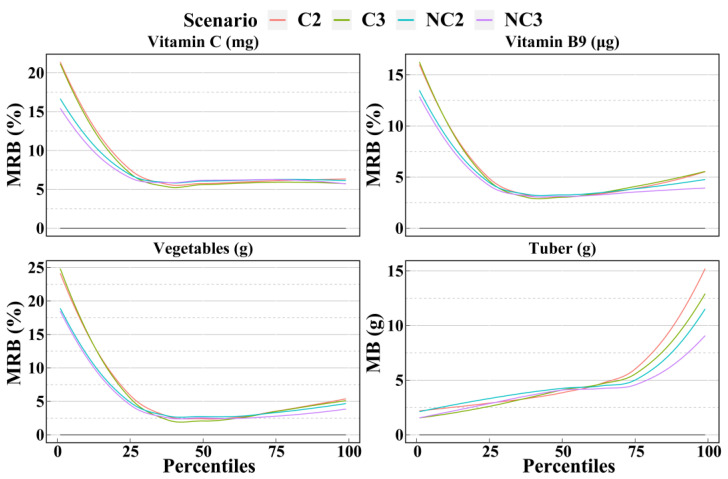
Smooth curves of the percentile (from 1st to 99th) intakes of dietary components in group 2 as calculated by the NCI method based on the 4 scenarios. Smooth curves of bias and relative bias for 10 dietary components calculated by the 4 scenarios are available in Supplementary Figure S3. C2 = 2 consecutive days; C3 = 3 consecutive days; NC2 = 2 non-consecutive days; NC3 = 3 non-consecutive days; MRB = Mean relative bias; MB = Mean bias.

**Figure 5:**
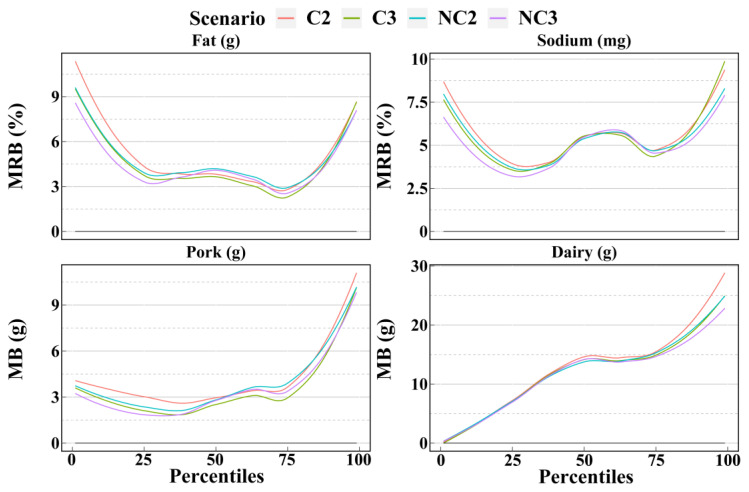
Smooth curves of the percentile (from 1st to 99th) intakes of dietary components in group 3 as calculated by the NCI method based on the 4 scenarios. Smooth curves of bias and relative bias for 11 dietary components calculated by the 4 scenarios are available in Supplementary Figure S4. C2 = 2 consecutive days; C3 = 3 consecutive days; NC2 = 2 non-consecutive days; NC3 = 3 non-consecutive days; MRB = Mean relative bias; MB = Mean bias.

Figure 3, Figure 4 and Figure 5 show the mean biases and mean relative biases of 4 scenarios. The 33 selected dietary components could be classified into 3 groups based on the accuracy from the 1st to the 99th percentiles estimated for the 4 scenarios. There were 12, 10, and 11 dietary components in each group, respectively. In the first group, the accuracy was significantly higher for scenarios with non-consecutive days than for scenarios with consecutive days and slightly higher for scenario NC3 than for scenario NC2. As shown in Figure 3, the order of the mean relative biases of protein and iron, from large to small, was scenarios C2, C3, NC2, and NC3. This order was more pronounced in the percentile outside the interquartile range. In the second group, only part of the percentile was in the above-mentioned order, while the accuracy of the remaining part did not differ significantly between the four scenarios. For example, for vitamin C and cereals, the accuracy of the 1st to 25th percentiles estimated by scenario NC3 was the highest, followed by scenario NC2; however, the accuracy of the 25th and 99th percentiles between the 4 scenarios was ambiguous and differed only slightly (Figure 4). In the third group, there was no clear trend in accuracy between the 4 scenarios, such as in cholesterol and eggs (Figure 5). In general, fact of whether the 24HRs were consecutive or non-consecutive was the main factor affecting the accuracy of dietary intake as estimated by the NCI method, rather than the number of 24HRs. In most cases, for percentiles, the accuracy of the non-consecutive 24HRs was higher than that of the consecutive 24HRs.

The mean biases and mean relative biases of the means and major percentiles of the intakes for each dietary component estimated by the NCI method relative to the true values can be found in Supplementary Table S1.

### 3.2. Equivalence Testing between Scenario NC2 and NC3

**Figure 6:**
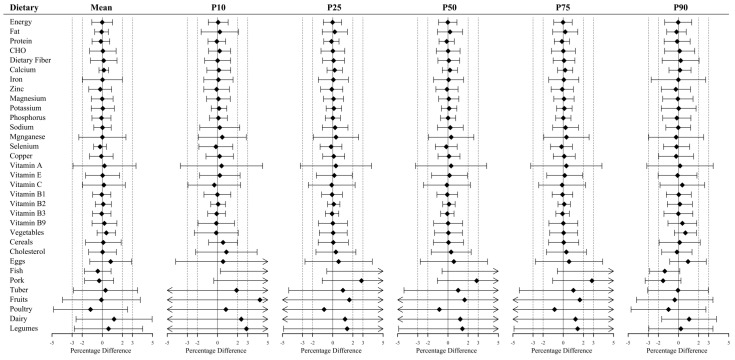
The 90% CI (Confidence Interval) for the percentage difference between scenario NC2 and NC3. The equivalence margins were 5% of scenario NC3 estimates. The equivalence was statistically significant if the 90% CI was within the range of −5% to 5% of the percentage difference. The specific values of the 90% CI are available in Supplementary Table S1. Mean = Equivalence test for means of scenarios NC2 and NC3; P10–P90 = Equivalence test for the corresponding percentiles of scenarios NC2 and NC3.

Figure 6 shows the 90% confidence interval (CI) for the percentage difference between scenario NC2 and NC3 when equivalence testing with equivalence margins of 5% of the estimates for the scenario NC3. It was clear that scenarios NC2 and NC3 estimated functionally identical means, 10th, 25th, 50th, 75th, and 90th percentiles for energy, vegetables, cereals, and all nutrients (*p* < 0.05). In addition, most of these dietary components had an error of 2% or less. Interestingly, the 90% CIs for cholesterol, iron, magnesium, vitamin A, and vitamin C were wider than those for other nutrients. For the other 8 foods, scenarios NC2 and NC3 estimated functionally identical means and 90th percentile (*p* < 0.05). However, for 10th, 25th, 50th, and 75th percentiles, the 90% CI for most foods exceeded the 5% of scenario NC3. Despite this, they were mostly within the 10% error range (data shown in Supplementary Table S2).

### 3.3. Comparison of Weekdays and Weekends in Scenario NC2

**Figure 7:**
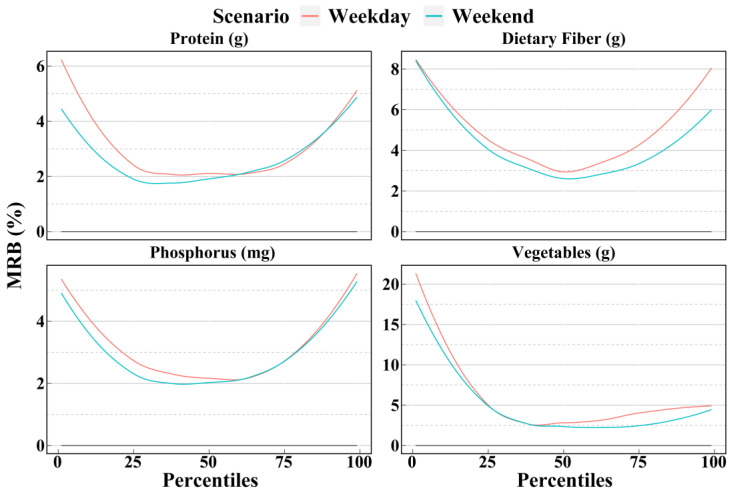
Smooth curves of the percentile (from 1st to 99th) intakes of dietary components in group 1 estimated by weekday and weekend of scenario NC2 based on the NCI method. Smooth curves of the bias and relative bias for each dietary component in group 1 as calculated by the four scenarios are available in Supplementary Figure S5. Weekday = Two non-consecutive weekdays; Weekend = Of the two non-consecutive days, one was a weekend, and the other was a weekday; MRB = Mean relative bias.

**Figure 8:**
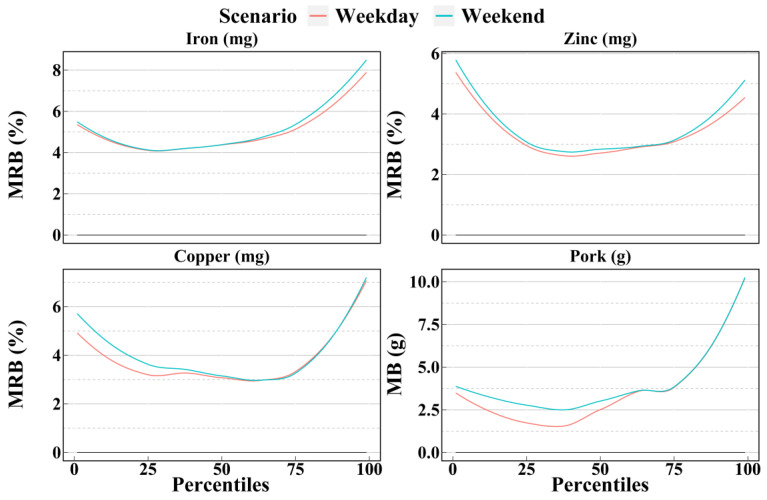
Smooth curves of the percentile (from 1st to 99th) intakes of dietary components in group 2 estimated by weekday and weekend of scenario NC2 based on the NCI method. Weekday = Two non-consecutive weekdays. Weekend = Of the two non-consecutive days, one was a weekend, and the other was a weekday; MRB = Mean relative bias; MB = Mean bias.

**Figure 9:**
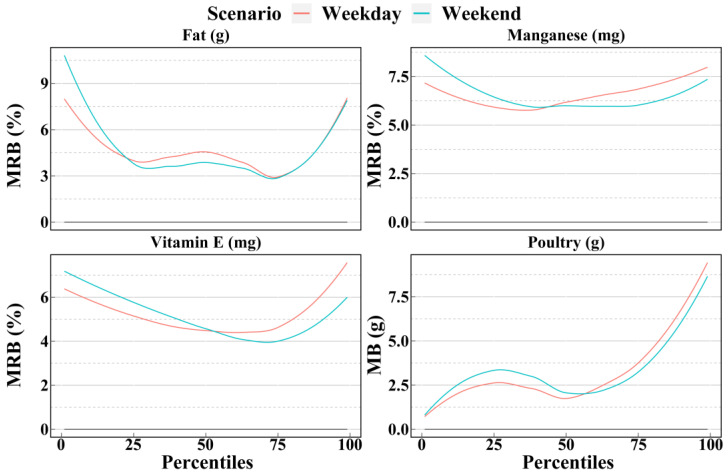
Smooth curves of the percentile (from 1st to 99th) intakes of dietary components in group 3 estimated by weekday and weekend of scenario NC2 based on the NCI method. Smooth curves of the bias and relative bias for each dietary component in group 3 calculated by the 4 scenarios are available in Supplementary Figure S6. Weekday = Two non-consecutive weekdays; Weekend = Of the two non-consecutive days, one was a weekend, and the other was a weekday; MRB = Mean relative bias; MB = Mean bias.

Figure 7, Figure 8 and Figure 9 show the mean biases and mean relative biases as estimated by weekdays and weekends in scenario NC2. The results can be divided into three group: weekend better, weekday better, and ambiguous. The first group includes 9 dietary components, as shown in Figure 7; weekends in scenario NC2 were more accurate than weekdays in scenario NC2 for most of the 1st to 99th percentiles. However, the second group included only four dietary components: iron, zinc, copper, and pork. As shown in Figure 8, in most cases, the accuracy estimated by weekdays was higher than that by weekends. A total of 18 dietary components were included in the third group. For these dietary components, the relationship between weekday and weekend accuracy varies with the percentile, and there was no clear trend. As shown in Figure 9, before the median, the accuracy of the weekday estimates of vitamin E was higher than that of the weekend; however, after the median, the results were reversed.

## 4. Discussion

Assessing the dietary intake of a population is a critical step in planning and evaluating interventions to address nutrient deficiencies or excess intakes and related health problems [32]. The 24HR method, a common method used in national nutrition surveys, is used to gather data on food intake that can be converted into daily nutrient intakes.

Collecting single 24-hour recall data from a large sample can accurately estimate the population-level average intake. However, it is inappropriate to use unadjusted single-day estimates to obtain the population distribution of the usual intake and calculate the prevalence of inadequacy/excess [16]. Therefore, multiple, repeated 24HRs are often used to address this limitation. However, different survey dates and repetition times can be combined into various forms. For example, NHANES uses 2 non-consecutive 24HRs, while CNHS uses 3 consecutive 24HRs [2,4]. The accuracy of the estimated dietary intakes and the cost of the survey vary with the different forms of the 24HR.

In our previous study, we compared the performance of the 24HR for 4 scenarios based on the within-person mean method, and we compared the accuracy of the NCI method and WPM method for four scenarios [11]. The results showed that the accuracy of the four scenarios calculated by the WPM method was similar, while the accuracy ranked from high to low was NC3, C3, NC2, C2, but the difference between those performed on consecutive and non-consecutive days was not significant. The number of survey days was the main factor affecting the accuracy of the 24HR. In addition, the results showed that the NCI method was always more accurate than the WPM method, regardless of the dietary components or the scenario. Dietary consumption varies according to the day, week, season, and person, and the biggest difference in nutrient intake was brought about by person, with greater variance within individuals than between individuals [9,33]. The NCI method can address this challenge via decomposing the total variance in transformed dietary intake into within-person variance (WPV) and between-person variance (BPV) and by eliminating the WPV component [34]. Hence, building on previous studies, the present study explored the form of the 24HR that simultaneously balanced accuracy and survey costs by comparing the performance of estimated dietary intakes based on 4 scenarios calculated by the NCI method and the effects of weekend/weekday and survey seasons.

The results showed that the precision was similar between the four scenarios in most cases. For most dietary components, there was a tendency for the distribution of bias to expand from the 5th to the 95th percentile, while the ranges of bias for the means and medians were similar, suggesting that the intakes fluctuated more across time for the high-intake group. This was consistent with a previous study for the 4 scenarios based on the WPM method, indicating that the intake calculation method did not affect the stability of the 24HR [11]. Furthermore, this variation affected the different forms of the 24HR equally although dietary intake varies seasonally and daily.

To overcome the seasonal and weekend effects, we used the average of the parameters calculated for all data sets for each scenario as the representative value of that scenario to compare the accuracy between the four scenarios. The results showed that, for different dietary components, the ranking of the accuracy of the four scenarios changed accordingly. In general, the order of accuracy from highest to lowest was: NC3, NC2, C3, and C2, with the difference between non-consecutive days and consecutive days being larger than the difference between 3 days and 2 days. As we expected, the continuity of the 24HRs was the main factor affecting the accuracy of dietary intake estimates, rather than the number of 24HRs. A previous study showed that, in the WPM method, the order of accuracy from highest to lowest was: NC3, C3, NC2, C2, which meant that the number of days was the main factor affecting accuracy, rather than whether the multiple, repeated 24HRs were consecutive [11]. These results were inconsistent with the present study, probably because the WPM method did not adjust for the total variance of dietary intake, whereas the NCI method was established to obtain the distribution of usual intake that was representative of the long-term based on short-term, repeated surveys. This indicated that the NCI method can eliminate the effect of days by calculating the distribution of dietary intake using only BPV components. However, since the effect of consecutive or non-consecutive survey days was not corrected, the NCI method recommended the use of non-consecutive days, which was consistent with the results of the present study [12].

Another aim of this study was to explore a form of 24HR that balances the costs and accuracy of the survey. Therefore, we performed an equivalence test between the scenario NC3 with the highest accuracy, and the scenario NC2 with the second highest accuracy because reducing the number of survey days was the most direct way to reduce costs. The results showed that for energy, nutrients, and frequently consumed foods, the errors of the mean and major percentiles between scenario NC2 and NC3 were mostly within 2%. Scenarios NC2 and NC3 estimated functionally identical means of foods consumed episodically, while for some percentiles, the errors between the two scenarios were mostly in the range of 5% to 10%.

The above results indicated that, for most dietary components, scenario NC2 was an acceptable substitute for scenario NC3. The problem of low accuracy in the foods consumed with low frequency can be addressed by including the food intake frequency (FFQ) as a covariate in the model. The scenarios share a common problem in estimating episodically consumed foods, namely producing a large error relative to the true values, which can be addressed by including food consumption frequency as a covariate in the model [13]. A previous study has shown that scenarios C3 and NC2 as based on the WPM method produced functionally identical means and medians, but there were larger errors between the two scenarios for the major percentiles, so some accuracy was lost by using scenario NC2 instead of C3 [11]. The results of the present study showed that the NCI method can reduce the difference between scenario NC2 and NC3 as compared to the WPM method, thus potentially reducing costs by reducing the number of survey days while maintaining accuracy. Then, we further divided scenario NC2 into two scenarios, weekday (both weekdays) and weekend (one weekday and one weekend) and compared the accuracy between the weekday and weekend scenarios. The results showed that the estimates for 9 dietary components calculated by the weekend scenario were more accurate than those calculated by the weekday scenario, while the opposite could be observed only for 4 dietary components. In most cases, the relationship between the accuracy between the two scenarios shifted with percentile and had no fixed trend. A previous study has shown that weekends tended to consume more energy than weekdays, and that the type of food consumed changed on weekends as compared to weekdays [9]. The NCI method was also developed with corresponding functions to correct for the weekday/weekend effect. Therefore, in conjunction with our findings, it may be more reasonable to use two non-consecutive 24HRs that include a weekday and a weekend. The study did not compare the accuracy across seasons because, in practice, it is difficult to standardize the times for conducting surveys at each survey site.

Based on the above results, we suggest that two non-consecutive 24HRs consisting of a weekend and a weekday using the NCI method can meet the requirements of high accuracy and low costs, and that is also the fewest number of survey days required to obtain usual intake. One way to further reduce the costs and subject burden may be to conduct a second repetition of the survey with only a part of the sample. A previous study showed that replication rates of a second 24HR greater than 40% did not lead to significant impairment in precision, and that impairment was reduced when the sample was increased, suggesting that choosing an appropriate replication rate to estimate dietary intake may help reduce costs [35]. There are many studies demonstrating the feasibility of online 24-h reviews, and their use can also reduce survey costs and subject burden [36,37]. The vehicles for implementing the 24HR method are diverse and can be used flexibly in actual investigations.

One of the strengths of this study was the use of 28 24HR, covering the 4 seasons, as a reference standard, rather than the simulated true dietary intake. The second strength was that we considered all possible combinations of 24HRs from different seasons and different days of the week. Additionally, we included 33 dietary components to comprehensively compare the performance of different forms of 24HRs involving energy, nutrients, and foods. However, there were some limitations. First, the reasons why different dietary components have different results were not further explored in this study. Second, the population of this study was Chinese adults, aged 18–60 years, so caution is needed when extrapolating the results.

## 5. Conclusions

In the NCI method, the continuity between multiple 24HRs is the main factor affecting the accuracy of the estimates, while the number of 24HRs has little effect on the accuracy. The precision is similar between the 4 forms of 24HR, while the order of accuracy varies across dietary components. In most cases, 3 non-consecutive 24HRs obtained the highest accuracy, followed by 2 non-consecutive 24HRs. For energy, nutrients, and frequently consumed foods, 2 non-consecutive 24HRs can replace 3 non-consecutive 24HRs to make functionally equivalent estimates. In addition, two non-consecutive 24HRs consisting of a weekend and a weekday performed better in estimating dietary intake. Therefore, we propose the use of 2 non-consecutive 24HRs, consisting of a weekend and a weekday, to collect data in large-scale nutrition surveys, and then to correct for within-person variance by the NCI method to improve accuracy while reducing survey costs and subject burden. However, this approach yielded great biases in estimating the intakes of foods consumed episodically, and further research is needed to determine how to reasonably collect food consumption frequency information under this approach so as to address this limitation.

## Figures and Tables

**Table 1:** Data sets generated from four scenarios over the seven consecutive survey days.

Scenarios	Total	Only Weekdays	Only Weekends	Weekdays and Weekends
C2	6	4	1	1
C3	5	4	0	1
NC2	15	6	0	9
NC3	10	1	0	9

Weekdays—from Monday to Friday; Weekends—Saturday and Sunday.

## Data Availability

The data presented in this study are non-public.

## Supplementary Materials

The following supporting information can be downloaded at: https://www.mdpi.com/article/10.3390/nu14132740/s1: Figure S1: Boxplot of bias and relative bias of intake calculated by 4 scenarios based on the NCI method; Figure S2: Mean bias and mean relative bias of the percentile intakes of dietary components in group 1 calculated by the NCI method based on the four scenarios; Figure S3: Mean bias and mean relative bias of the percentile intakes of dietary components in group 2 calculated by the NCI method based on the four scenarios; Figure S4: Mean bias and mean relative bias of the percentile intakes of dietary components in group 3 calculated by the NCI method based on the four scenarios; Figure S5: Mean bias and mean relative bias of the percentile intakes of dietary components in group 1 estimated by weekday and weekend within scenario NC2 with the NCI method; Figure S6: Mean bias and mean relative bias of the percentile intakes of dietary components in group 3 estimated by weekday and weekend within scenario NC2 with the NCI method; Table S1: The mean bias and mean relative bias of estimates obtained with each scenario; Table S2: The 90% CI for the percentage difference between scenario NC2 and NC3.
